# Biochemical and Clinical Assessments of Segmental Maxillary Posterior Tooth Intrusion

**DOI:** 10.1155/2017/2689642

**Published:** 2017-02-22

**Authors:** Jintana Tasanapanont, Tanapan Wattanachai, Janya Apisariyakul, Peraphan Pothacharoen, Siriwan Ongchai, Prachya Kongtawelert, Marit Midtbø, Dhirawat Jotikasthira

**Affiliations:** ^1^Department of Orthodontics and Pediatric Dentistry, Faculty of Dentistry, Chiang Mai University, Chiang Mai, Thailand; ^2^Department of Biochemistry, Faculty of Medicine, Chiang Mai University, Chiang Mai, Thailand; ^3^Department of Clinical Dentistry-Orthodontics, Faculty of Medicine and Dentistry, University of Bergen, Bergen, Norway

## Abstract

*Objective*. To compare chondroitin sulphate (CS) levels around maxillary second premolars, first molars, and second molars between the unloaded and the loaded periods and to measure the rates of intrusion of maxillary posterior teeth during segmental posterior tooth intrusion.* Materials and Methods*. In this prospective clinical study, 105 teeth (from 15 patients exhibiting anterior open bite and requiring maxillary posterior tooth intrusion) were studied. Competitive ELISA was used to detect CS levels. Dental casts (during the unloaded and loaded periods) were scanned, and posterior tooth intrusion distances were measured.* Results*. During the unloaded period, the median CS levels around maxillary second premolars, first molars, second molars (experimental teeth), and mandibular first molars (negative control) were 0.006, 0.055, 0.056, and 0.012 and during the loaded period were 2.592, 5.738, 4.727, and 0.163 ng/*μ*g of total protein, respectively. The median CS levels around experimental teeth were significantly elevated during the loaded period. The mean rates of maxillary second premolar and first and second molar intrusion were 0.72, 0.58, and 0.40 mm/12 weeks, respectively.* Conclusions*. Biochemical and clinical assessments suggested that the segmental posterior tooth intrusion treatment modality with 50 g of vertical force per side was sufficient.* Trial Registration*. The study is registered as TCTR20170206006.

## 1. Introduction

In an anterior open bite malocclusion, maxillary and mandibular anterior teeth are not in contact, and vertical overlap does not exist when clinically examined from the frontal view. Orthodontic treatment for anterior open bite includes anterior tooth extrusion, posterior tooth intrusion, or a combination of the two. The stability of anterior tooth extrusion is questionable; therefore, posterior tooth intrusion is preferable. Skeletal open configuration is also caused by excessive vertical development of both maxillary and dentoalveolar parts, especially in the posterior tooth region. In growing patients, treatment modalities for anterior open bite are aimed at reducing, redirecting, impeding, or modifying the patient's vertical growth. In nongrowing adult patients, on the other hand, absolute intrusion of the posterior part of the dentition (both maxillary and mandibular) might be required to improve this malocclusion. However, the severity of anterior open bite should be considered during conventional orthodontic treatment planning. Orthodontic-surgical treatment is appropriate for severe skeletal open bite.

Several investigations [1, 2] have attempted to quantify the optimal force required for orthodontic maxillary posterior tooth intrusion. For maxillary molar intrusion, the force magnitude ranges from 100 g to 200 g and from 200 g to 400 g for segmental maxillary posterior tooth intrusion. Melsen and Fiorelli [3] suggested 50 g of force for single molar intrusion in adults. S. Kato and M. Kato [4] found that 100 g of force was insufficient for segmental posterior tooth intrusion, but smooth progressive intrusion was achieved when the force levels were increased to 300 g per side. This variation might be explained by the different intrusion techniques. There is a consensus that the magnitude of force, acting upon the tooth and being responsible for the tooth movement, must be carefully controlled. Heavy intrusive force has shown reduction of pulpal blood flow and risk of pulp necrosis [5]. Therefore, it is important to investigate orthodontic force magnitude for intruding maxillary posterior teeth, in order to treat anterior open bite without collateral effects.

During orthodontic treatment, the applied forces produce a distortion of the periodontal ligament, resulting in alterations in cellular and cytoskeletal configuration. Chondroitin sulphate (CS) has been categorized as a tissue breakdown product of bone metabolism. CS constitutes approximately 94% of the glycosaminoglycans in human alveolar bone [6]. The high quantity of CS in human alveolar bone might be the main source of CS in gingival crevicular fluid (GCF). Orthodontic force causes considerable localized remodeling of the alveolar bone and brings about changes in the CS levels in GCF. Intachai et al. [7] monitored changes in CS around immobile miniscrew implants, during the unloaded and loaded periods, and concluded that CS could be detected and might be used as biomarkers for assessing alveolar bone remodeling around miniscrew implants during orthodontic loading. Furthermore, our previous study [8, 9] reported increases in CS levels in GCF around orthodontically moved canines. Consequently, alveolar bone remodeling resulting from orthodontic force would be expected to raise the CS levels found in GCF.

Our objectives were to compare CS levels in GCF around experimental maxillary second premolars and first and second molars between the unloaded and loaded periods and to assess rates of intrusion of maxillary posterior teeth and overbite changes during segmental maxillary posterior tooth intrusion.

## 2. Materials and Methods

### 2.1. Subjects

This prospective clinical study was approved by the Human Experimentation Committee of the Faculty of Dentistry, Chiang Mai University, Thailand (number 19/2558). Informed consent was obtained from all patients. One hundred and five teeth from 15 patients (1 male and 14 females; mean age 20.2 ± 2.8 years; range 15.0–30.5 years; with skeletal open configuration and anterior open bite), who required orthodontic maxillary posterior tooth intrusion, were recruited. All patients, with anterior open bite, who were treated at Orthodontic Division, Faculty of Dentistry, Chiang Mai University between October 2013 and December 2014, who met the following inclusion criteria were included in the trial: (1) healthy with no systemic medical conditions and no routine medications; (2) healthy periodontium, no bleeding on probing and probing depth of 3 mm or less at all teeth and no radiographic evidence of bone loss; (3) nonsmoker; (4) no pregnancy (women); and (5) no previous experience of orthodontic treatment.

### 2.2. Experimental Design

The experimental design was divided into two phases.

*Phase I: The Unloaded Period*. An assessment of the general status of the patients and informed consent were obtained. Prior to the orthodontic loading, GCF was collected from the maxillary second premolar, first and second molars (experimental), and right mandibular first molar (negative control). Oral and gingival health was monitored and maintained throughout the entire study.

**Figure 1:**
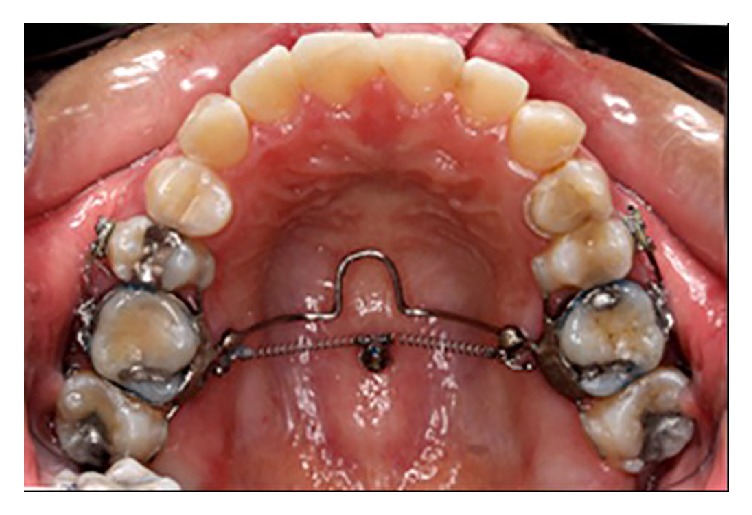
Intraoral photograph showing the segmental posterior tooth intrusion (group-of-six) treatment modality using an intrusion transpalatal arch with tags and two nickel-titanium closed coil springs (100 g of force each). One titanium miniscrew implant (2.0 mm diameter, 6.0 mm length) was placed in the midpalatal area.

One titanium miniscrew implant (2.0 mm diameter, 6.0 mm length) (Dual Top Anchor System, Jeil Medical Corporation, Seoul, Korea) was placed in the midpalatal area, under local anesthesia, and its placement position corresponded to the maxillary first molar position; after that the impression was taken for an intrusion transpalatal arch (TPA). The miniscrew implant was monitored for four weeks before force application. An intrusion TPA was fabricated and soldered to the right and left maxillary first molar bands. In order to achieve the segmental posterior tooth intrusion, brackets and tubes (0.018 Roth prescription, Gemini series, 3M Unitek, Monrovia, California, USA) were bonded on the buccal surface of the maxillary second premolars and second molars, and sectional 0.017 × 0.025 inch stainless steel wires were passively inserted and tied on both sides (Figure 1).

*Intrusion Transpalatal Arch Fabrication*. The standard TPA design was customized as follows.

**Figure 2:**
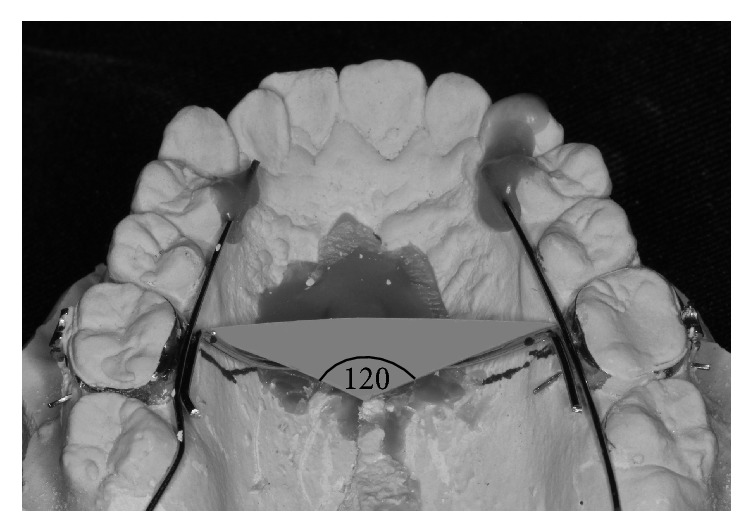
The angle between the two force vectors of the two nickel-titanium closed coil springs was controlled to be 120°.

1. Both right and left maxillary first molars were banded.
2. A 1.0 mm diameter stainless steel wire was soldered to both maxillary first molar bands, in order to provide rigidity for the TPA, and two crimpable hooks (Tomy, Tokyo, Japan) were soldered to the TPA. The angle between the two closed coil springs was controlled to be 120°. (Figure 2). Tags were fabricated to create group-of-six segmental maxillary posterior tooth intrusion.
3. The TPA was offset, approximately 5.0 mm, from the tissue surface of the hard palate, especially in the midpalatal area, to allow for its gradual vertical displacement toward the palatal tissue surface during maxillary molar intrusion. It was also required to have adequate clearance from the lateral aspects of the palatal alveolar tissue.

**Figure 3:**
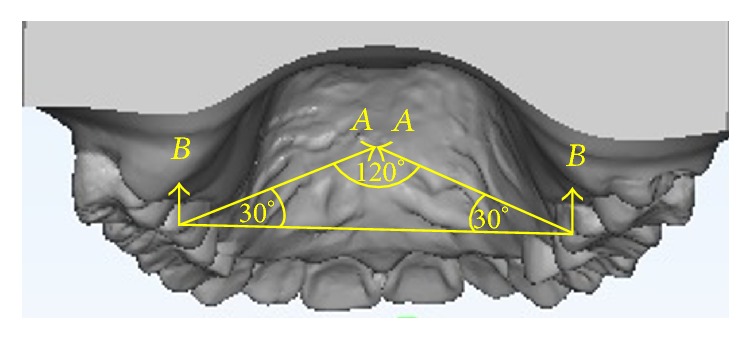
The force magnitude (*A*) of each nickel-titanium closed coil spring was 100 g per side. The magnitude of the resultant intrusive force (*B*) for either right or left side was equal to 50 g.

*Phase II: The Loaded Period*. Four weeks after miniscrew implant placement, two nickel-titanium closed coil springs were connected between the midpalatal miniscrew implant head and both right and left soldered hooks on the TPA in order to generate orthodontic intrusive force. The force magnitude (*A*) from each nickel-titanium closed coil spring was calibrated and controlled to be 100 g per side. The angle between the two nickel-titanium closed coil springs was controlled to be 120°. The magnitude of the resultant intrusive force (*B*), for either right or left side, which was calculated from the formula: *B* = *A*(sin⁡30°), was equal to 50 g (Figure 3).

**Figure 4:**
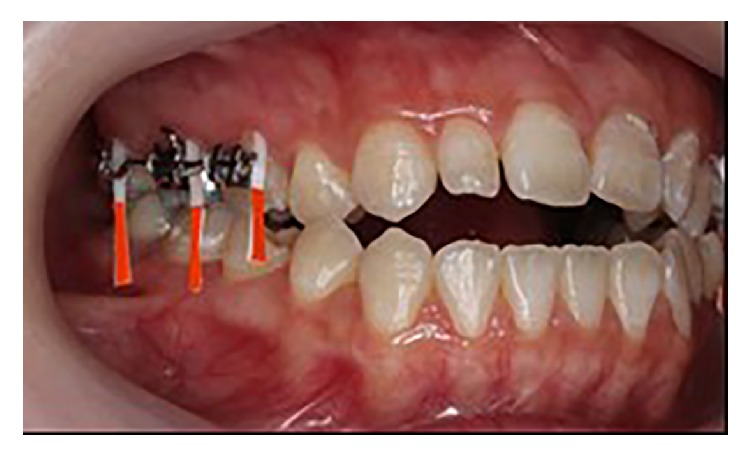
Periopaper strips were placed into the mesiobuccal sulcus for GCF sample collection.

*Biochemical Assessment of Segmental Maxillary Posterior Tooth Intrusion*. GCF samples were collected from patients every week from week 0 (baseline data) to week 8. In order to collect GCF, the experimental and control sites were isolated from saliva and gently air-dried. All samples were collected using Periopaper® (Oraflow, Plainview, New York, USA) strips inserted into the gingival sulcus at the mesiobuccal area of the experimental and control teeth (Figure 4). The reasons the mesiobuccal sulcus area was selected were that this area had favorable cleaning access by typical self-administered home care procedures and that easy accessibility to this surface was likely to produce more reproducible sampling than taking samples from the distobuccal or palatal surfaces of the teeth [16]. Bilateral maxillary posterior teeth were used for the measurement CS levels. The volume of GCF collected from the last 2.0 mm of each paper strip was 0.1 *μ*L [7]. Competitive ELISA with WF6 monoclonal antibody was used to detect CS levels in the collected GCF. Two related but distinct chondroitin sulphate mimetope octasaccharide sequences were recognized by monoclonal antibody WF6 [17]. The CS levels in all samples were measured in nanograms per microgram of total protein content (ng/*μ*g).

*Competitive ELISA with WF6 Monoclonal Antibody*. Shark PG-A1 fraction (100 *μ*L/well) was absorbed overnight at room temperature on microtiter plates (Maxisorp®, Nunc, Roskilde, Denmark) in 0.2 M sodium carbonate, pH 9.6. The wells were washed three times with Tris-IB buffer and blocked with Bovine serum albumin (BSA) 1% (w/v) 150 *μ*L/well in incubating buffer (Tris-IB) for 1 h at 37°C and washing. The coated wells were incubated with Shark PG-AlDl fraction: range 39.06–10,000 ng/mL and a mixture of mAb WF6 (1 : 100) for 1 h at 37°C. After washing, the anti-mouse IgM-specific peroxidase conjugated with horseradish peroxidase (100 *μ*L/well; 1 : 2000) was added and incubated for 1 h at 37°C. Then, the plates were washed three times, and orthophenylenediamine substrate was added and incubated for 20 minutes at 37°C to allow the color to develop. The colored reaction product was quantified in an ELISA reader Titertek Multiskan (Flow Laboratories, Meckenheim, Germany) by absorbance ratio at 492/690 nm.

Total protein concentration was determined by the Bio-Rad protein assay (Bio-Rad Laboratories, Hercules, California, USA) based on the Bradford dye-binding procedure.

**Figure 5:**
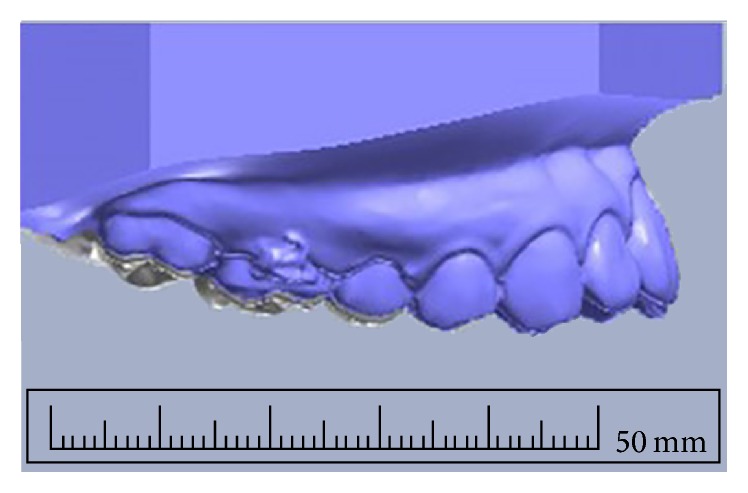
Clinical assessment by superimposition of 3D scans of before (gray) and after (blue) intrusion dental casts.

*Clinical Assessment of Maxillary Posterior Tooth Intrusion Distance*. Coil springs were removed and then alginate impression was taken for the fabrication of study models. Dental casts were made prior to posterior tooth intrusion (as baseline data) and at the twelfth week of posterior tooth intrusion (as experimental data). Rate of molar intrusion was extended further for four additional weeks (up to 12 weeks) in order to show a clearer picture of maxillary posterior tooth intrusion. Dental casts were scanned using an OrthoAnalyzer (3Shape, Copenhagen, Denmark) to obtain stereolithography data. The stereolithography images of the untreated canines and incisors provided a reference for superimposition (Figure 5).

The posterior tooth intrusion distance was measured by the ImageJ scientific image processing program [18]. The measurements included the average vertical intrusion distance from the cusp tips of the orthodontically moved maxillary second premolar and first and second molars. For maxillary molars, the mean intrusion distances were the means of the combination of intrusion distance of mesiobuccal and distobuccal cusps. Initial dental casts of upper and lower arches from each subject were expressed in the maximum intercuspidation position. The overbite values were measured in vertical distance between edge of upper and lower incisors, in millimeters. Twelfth weeks after maxillary posterior tooth intrusion, progressive models were taken and overbite changes were assessed.

*Statistical Analysis*. The data were analyzed using the Statistical Package for Social Sciences version 17.0 for Windows (SPSS Inc., Chicago, Illinois, USA). The differences between the CS levels during the unloaded and the loaded periods were determined using the Mann–Whitney *U* test. The results were considered statically significant at *P* < 0.05.

## 3. Results

### 3.1. Elevated Levels of CS (WF6 Epitope) in GCF around Maxillary Posterior Teeth during Segmental Maxillary Posterior Tooth Intrusion

**Figure 6:**
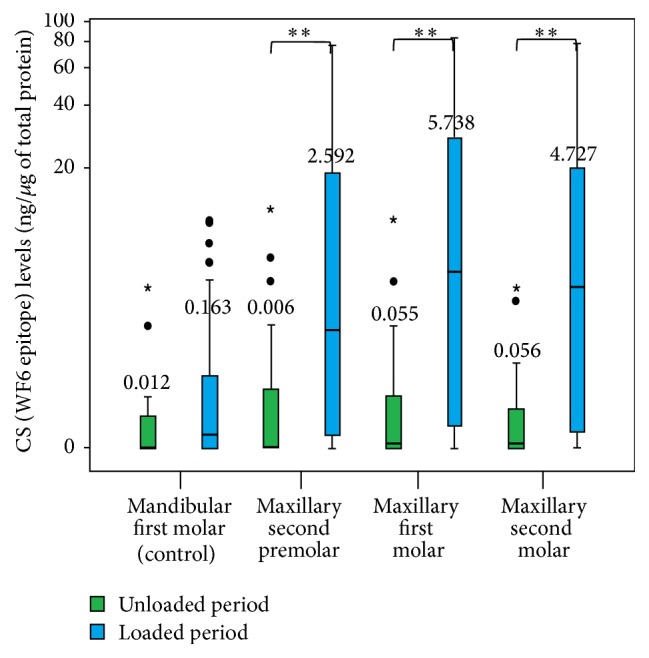
A boxplot graph of the chondroitin sulphate (CS) levels around the mandibular first molar (negative control) and the maxillary second premolar and first and second molars (experimental) during the unloaded and loaded periods. The small circles represent outlier values, and small asterisks represent extreme values. ^*∗∗*^Significant difference: *P* < 0.05.

During the unloaded period, the median CS levels around the mandibular right first molars (negative control) were 0.012 ng/*μ*g, and those around the maxillary second premolars and first and second molars (experimental) were 0.006, 0.055, and 0.056 ng/*μ*g of total protein, respectively. During the loaded period, the median CS levels around the mandibular right first molars (negative control) were 0.163 ng/*μ*g, and those around the maxillary second premolars and first and second molars (experimental) were 2.592, 5.738, and 4.727 ng/*μ*g of total protein, respectively. The median CS level around the mandibular right first molars (negative control) during the loaded period was not significantly different from that during the unloaded period, but the median CS levels around the maxillary second premolars and first and second molars (experimental) during the loaded period were significantly greater than those during the unloaded period (*P* < 0.05) (Figure 6).

**Figure 7:**
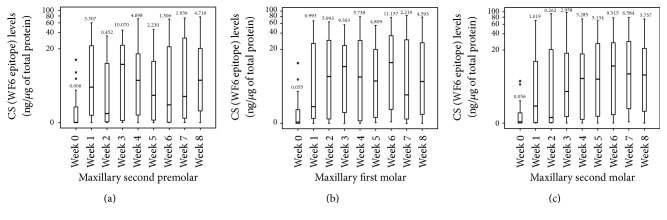
A boxplot graph of the chondroitin sulphate (CS) levels around the maxillary second premolar (a), first molar (b), and second molar (c) during the unloaded period (week 0) and the loaded periods (week 1 to week 8). The small circles represent outlier values, and small asterisks represent extreme values.

**Figure 8:**
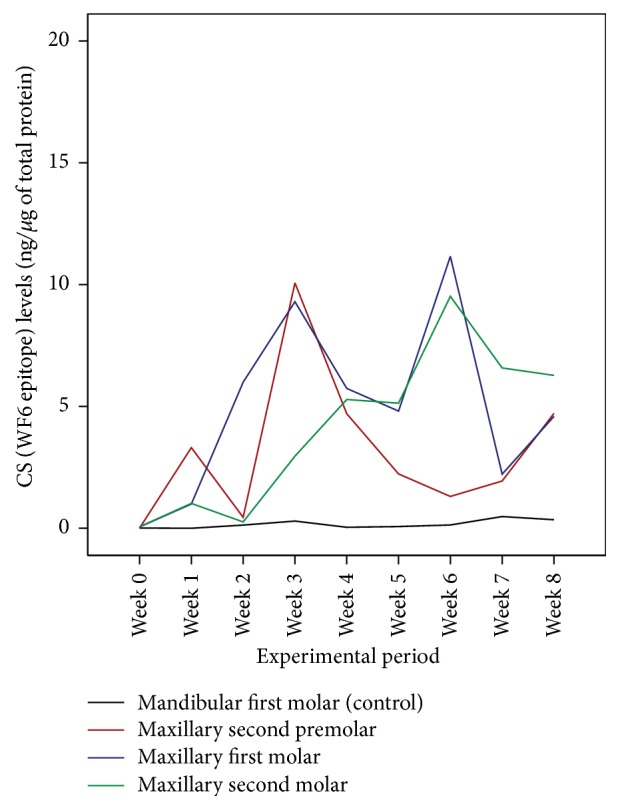
A profile graph of the CS levels in a subject during the unloaded period (week 0) and the loaded periods (week 1 to week 8).

The median CS levels around the maxillary second premolars and first and second molars from week 0 (unloaded) to week 8 are shown in Figure 7. The median CS levels around the experimental teeth from each week (during the 8-week loaded period) were significantly greater than those from week 0 (the unloaded period) (*P* < 0.05). It should be noted that, during the loaded period (week 1 to week 8) of the experimental teeth, the median CS levels showed a cyclical pattern (Figure 8).

### 3.2. Rates of Intrusion of Posterior Teeth during Segmental Maxillary Posterior Tooth Intrusion and Anterior Overbite Change

The mean rates of intrusion of maxillary second premolar and first and second molar intrusion were 0.72, 0.58, and 0.40 mm/12 weeks, respectively (average of maxillary posterior teeth = 0.19 mm/month). The rates of intrusion ranged from 0.21 mm to 1.53 mm/12 weeks for second premolars, 0.3 mm to 1.13 mm/12 weeks for first molars, and 0 mm to 0.84 mm/12 weeks for second molars. The anterior overbite was improved in all cases, and the mean of anterior overbite changes was 1.12 mm within 12 weeks.

## 4. Discussion

The standardization of the intrusion treatment modality, the amount and vector of intrusive force, and the location of the miniscrew placement site were controlled in our study. For our treatment modalities for maxillary posterior tooth intrusion, the force magnitude (as low as 50 g per side) was lower than previously recommended (Table 1) [10–15].

The benefit of our maxillary molar intrusion treatment modality was that the maxillary posterior teeth were intruded using only one midpalatal miniscrew implant in combination with a TPA. The midpalatal suture is the preferred site for miniscrew implant placement because of its ease of miniscrew placement, its good bone thickness and density, and its lack of vital anatomical structures. The TPA was applied at the palatal site to prevent palatal tipping when intrusive force was applied by nickel-titanium closed coil springs. However, miniscrew implants and a TPA might irritate the tongue in patients with a low palatal vault. An alternative site for miniscrew implant placement for maxillary posterior tooth intrusion is the interradicular area, either buccally or palatally [19]. A disadvantage of the interradicular area placement is the risk of root damage. A recent study that evaluates skeletal and dental changes after using miniscrews with TPA and elastomeric chain for intrusion of the maxillary molars by Hart et al. [20] found that an average of 0.2 mm molar intrusion and 1.1 mm increase in overbite. However, they did not standardized force magnitude, location of miniscrews, and vector of traction but our research controlled force magnitude, miniscrew placement, and force vector of two closed coil springs.

Throughout the study periods, the median CS levels around mandibular first molars (negative control) were relatively low and showed no cyclical pattern. The reasons the right mandibular first molars were used as negative control teeth were that mandibular arch was not bonded during experimental period and that it justified easy access and repetition. During the unloaded periods, CS levels around the experimental teeth were relatively low, whereas during the loaded periods, the CS levels were elevated and showed a cyclical pattern similar to that reported in our previous study [8]. The raised CS level showed alveolar bone remodeling activities during maxillary posterior tooth intrusion. The CS levels around the maxillary second premolars were elevated to the peak levels at the 3rd week and 8th weeks, those around the first molars peaked at the 3rd and 6th weeks, and those around the maxillary second molars at the 4th week and 6th weeks. A possible explanation may be that, with our particular posterior tooth intrusion treatment modality, the intrusive force was probably directed toward the first molar and second premolar areas. A clinical research design for further investigation which changes the palatal miniscrew implant placement location, either posteriorly or anteriorly in order to determine the intrusive force direction, may elucidate the effect of force direction during posterior tooth intrusion treatment.

Our research protocol was designed to monitor CS levels in an experimental period of eight weeks in accordance with our previous study [8]. However, measuring the rate of segmental posterior tooth intrusion was extended to the twelfth weeks in order to show a clearer picture of tooth intrusion due to the slow nature of orthodontic tooth movement. The rate of molar intrusion in our study was similar to that in other studies where the rate of tooth movement was calculated per month [11, 14]. The rate of molar intrusion in our study was less than that reported in the study of Scheffler et al. in which 150 g of intrusive force was used, and 2.3 mm of maxillary molar intrusion (within six months) was reported [21]. The different rates of molar intrusion might be due to different force magnitudes per unit of root surface area. However, during orthodontic tooth intrusion, heavy forces should be avoided, given the fact that the force is distributed over a small area around the root apex. The high magnitude of intrusion force can significantly influence the amount of root resorption, especially in open bite cases because of the demonstrated higher root resorption than in normal cases [22]. To prevent root resorption, light force during intrusion is preferable.

Furthermore, our study showed 50 g per side could effectively induce biological changes and justify maxillary segmental posterior tooth intrusion. Our study suggested that it might be useful to demonstrate force per unit of root surface area during segmental posterior tooth intrusion. The average root surface areas of the maxillary second premolar and first molar and second molar have been reported to be 254, 533, and 450 mm^2^, respectively [23]. Accordingly, the intrusive force magnitude per unit of root surface area used for segmental posterior tooth intrusion in this study was only 0.04 g/mm^2^. However, the average root surface areas which were cited in those studies were Caucasian norms. The root surface areas of Thai populations may be different from those of Caucasian ones; therefore, the root surface area of various populations, as well as various facial patterns and configurations, should be further investigated to determine proper intrusive force magnitudes for maxillary posterior tooth intrusion. Long-term stability of this treatment modality should also be further investigated.

## 5. Conclusions

Our particular miniscrew-assisted treatment modality for segmental maxillary posterior tooth intrusion using an intrusion transpalatal arch and two nickel-titanium closed coil springs (vertical force 50 g per side) caused detectable biochemical alveolar bone remodeling activities as revealed by raised CS levels in GCF around maxillary posterior teeth, by detected maxillary posterior tooth intrusion distances, and by improved anterior overbite.

## Figures and Tables

**Table 1:** Summary of articles on segmental maxillary posterior tooth intrusion.

Authors	Year	Type of study	Number	Age (yrs)	Method	Teeth	Force (g)	Intrusion rate (mm/month)	Overbite change
Abdullatif and Keles [10]	2001	Prospective	8	11.1–13.8	Headgear	Maxillary teeth	500	0.47	0.63
Meral and Yüksel [11]	2003	Prospective	16	9.5–13.5	Plate with magnetic	Maxillary and mandibular teeth	300	0.01 and 0.10	3.94
Erverdi et al. [12]	2004	Prospective	10	17.0–23.0	Miniplate and spring	Maxillary posterior teeth (4–7)	400	0.51	0.73
Erverdi et al. [13]	2006	Case report	1	14	Miniplate and spring	Maxillary posterior teeth (4–7)	400	0.51	1.29
Xun et al. [14]	2007	Prospective	12	14.3–27.2	Miniscrew and chain	Maxillary teeth	150	0.26	0.62
Foot et al. [15]	2014	Prospective	16	12.2–14.3	Miniscrew and spring	Maxillary posterior teeth (4–7)	500	0.59	0.61
Our study		Prospective	15	15.0–30.5	Miniscrew and spring	Maxillary posterior teeth (5–7)	50	0.19	0.37
